# Rab11 in Disease Progression

**Published:** 2015

**Authors:** Tanmay Bhuin, Jagat Kumar Roy

**Affiliations:** 1 *Cell and Developmental Biology Unit, Department of Zoology, The University of Burdwan, Golapbag-713104, India.*; 2 *Cytogenetics Laboratory, Department of Zoology, Banaras Hindu University, Varanasi-221 005, India.*

**Keywords:** Vesicle trafficking, Rab proteins, Ras superfamily, Rab11, disease progression

## Abstract

Membrane/protein trafficking in the secretory/biosynthetic and endocytic pathways is mediated by vesicles. Vesicle trafficking in eukaryotes is regulated by a class of small monomeric GTPases: the Rab protein family. Rab proteins represent the largest branch of the Ras superfamily GTPases, and have been concerned in a variety of intracellular vesicle trafficking and different intracellular signalling pathways. Rab11 (a subfamily of the *Ypt/Rab *gene family), an evolutionarily conserved ubiquitously expressed subfamily of Rab GTPases, has been implicated in regulating vesicular trafficking through the recycling of endosomes. Rabs have been grouped into different subfamilies based on the distinct unambiguous sequence motifs. Three members: Rab11a, Rab11b and Rab25 make up the Rab11 GTPase subfamily. In this review article, we describe an overview over Rab11 subfamily with a brief structural aspect and its roles in implicating different disease progression.

**Overview over Rab family proteins and Rab11 subfamily**

Rab proteins, the evolutionarily conserved small (21-25 kDa) monomeric GTP-binding molecules (GTPases) forming the largest branch of the Ras superfamily, have been found to be present from yeast to humans. These proteins participate in receptor cargo collection during transport vesicle formation and enable motor proteins to interact with membranes to drive vesicle motility, mediating the complex events of precise docking and fusion of transport vesicles with their targets. In addition to the aforesaid general functions, Rabs also act as key players in performing a variety of cellular functions, *viz.*, growth, protein trafficking and different signalling pathways. So far, over 70 Rab proteins are encoded by the vertebrates; while most of them are ubiquitously expressed but some of these are tissue-specific also. A variety of Rabs are defective and abnormally expressed in different diseases including cancers (1). Rabs are associated with cyclical activation and inactivation in the form of GTP-bound/active state-regulated by upstream regulators known as GEFs (guanine nucleotide exchange factors) and GDP-bound/inactive state- regulated by GAPs (GTPase activating proteins) between the membranes and cytosol, respectively. In their active states, Rab proteins are associated with the cytosolic face of membranous compart-ments and act together with downstream effector proteins carrying out their biological functions (2). Yeast has a relatively simple endomembrane system and contains only 11 Rab proteins. In humans, the numbers of Rab proteins have progressively been increased along with the evolution to almost 70 family members, comparable to an increase in the complexity of vesicular trafficking system (3-4). In addition, around 33 Rabs in *Drosophila melanogaster *(5), 29 in *Caenorhabditis elegans*, 57 in* Arabidopsis thaliana* (6-7) have also been characterized so far.

Rab11, an evolutionary conserved ubiquitous-ly expressed subfamily of the *Ypt/Rab *gene family, is associated with recycling endosomes (REs) and has been implicated in regulating vesicular trafficking through recycling of endosomal compartment (REC) and early endosomes (EEs) to the trans- Golgi network (TGN) and plasma membrane through recycling pathways (8-10). *Drosophila *has a single *Rab11* gene whereas, the vertebrate Rab11 subfamily consists of three members *viz.,* Rab11a, Rab11b and Rab25, and they have been characterized as regulators of REs in both polarized and non-polarized cells which are encoded by distinct genes (11-13). This review focuses on a brief structural description of Rab11, and its roles in implicating disease progression.

**Structure of Rab11**

Rab GTPases undergo post-translational modifications for its functional activity. Rab11a terminates with isoleucine at its C-terminus, suggesting that it may be geranylgeranylated. Studies showed that Rab11a can be modified *in vitro,* by different prenyl groups: farnesyl and geranylgeranyl (14), whereas *in vivo,* it is exclusively modified post-translationally only by geranylgeranyl groups indicating that there must be other determinant(s) that are essential for prenyltransferase recognition (15). Pasqualato et al. 2004 (16) have reported the crystallographic analysis of the GDP/GTP cycle of human Rab11a. The crystal structures indicate that the nucleotide- sensitive switch 1 and 2 regions differ from those of other Rab proteins. In Rab11-GDP, they contribute to a close crammed symmetrical dimer, which may associate with membranes in the cell and allow Rab11 to undergo GDP/GTP cycles without recycling to the cytosol. The structure of active Rab11 delineates a three-dimensional site that includes switch 1 and is separated from the site defined by the Rab3/Rab-philin interface. It is proposed to form a novel interface for a Rab11 partner compatible with the simultaneous binding of another partner at the Rab- philin interface. Mutation of Ser 29 to Phe in this epitope resulted in morphological modifications of the recycling compartment that are distinct from those induced by the classical dominant-negative and constitutively- active Rab11 mutants.

**Rab11 dysfunction and diseases**

Rab11 has been implicated in a number of patho-physiological diseases (Table 1), which are summarized below:

**Table 1:** Diseases associated with Rab11 subfamily members

**Disease name**	**Subfamily member**	**References**
Alzheimer’s disease	Rab11a/Rab11b	17- 20
Huntington’s disease	Rab11a/Rab11b	21- 27
Type 2 diabetes	Rab11a/Rab11b	28, 29
Cancer (breast, colon, lung, ovarian, renal, endometrial, prostate, bladder)	Rab25	30- 50

**Neurodegenerative diseases**

Most of the neurodegenerative diseases are characterized by synaptic dysfunction; neuronal loss and cognitive or behavioural anomalies. Rab11 has been implicated in the following neuro-degenerative diseases:

**Alzheimer’s disease (AD)**

It is the most common form of dementia and has been characterized by the cerebral deposition of β-amyloid (Aβ) peptides as amyloid plaques which are generated from amyloid precursor protein (APP) by β and γ secretases. APP and the secretases are membrane associated proteins (17-18). Aggregation of Aβ peptides leads to synaptic disturbance and neurodegeneration in AD. A portion of endocytosed Aβ is transported through Rab11-positive recycling vesicles. Cellular accu-mulation of Aβ is enhanced after inhibiting Aβ recycling pathway by using a constitutively-active Rab11. Inhibition of lysosomal enzymes results in the Aβ accumulation and aggregation (19). An RNAi screen using human Rab GTPases, identified that Rab11 has been implicated in the recycling of β-secretase to the plasma membrane and thereby affecting Aβ production. Exome sequencing exposed an important genetic relationship of Rab11a with late-onset AD, and network analysis identified Rab11a and Rab11b as components of the late-onset AD risk network. This suggests a causal link between Rab11 and AD indicating trafficking pathways regulating Aβ levels and unravels the molecular complexity underlying AD (20).

**Huntington disease (HD)**

It is characterized by the presence of nuclear and cytoplasmic inclusions, striatal neuronal loss, and gliosis. Disruption of axonal transport within long, narrow-caliber axons results in the accumulations causing cell death and ultimately neuronal dysfunction. Synaptic dysfunction in a *Drosophila* model of HD can be prevented by enhanced neuronal expression of Rab11. Inhibition of Rab11 function in fibroblasts of HD patients has been observed to perturb vesicle formation from recycling endosomes. Rab11 is involved in synaptic dysfunction prior to the onset of HD symptoms, with the aim of finding a possible early intervention to disease progression. Rab11 ameliorates synaptic dysfunction due to expression of mutant huntingtin (the causative protein in HD) by normalizing synaptic vesicle size, which consequently ameliorates locomotor deficits in *Drosophila *larvae (21). The HD mutation causes polyglutamine expansion in huntingtin protein (Htt) and neuro-degeneration. Htt interacts with a complex containing Rab11-GDP and is involved in the activation of Rab11, which functions in endosomal recycling and neurite growth and long term potentiation. Rab11-GDP undergoes nucleotide exchange to Rab11-GTP for its activation. Striatal membranes of HD 140Q/140Q knock-in mice are impaired in supporting conversion of Rab11-GDP to Rab11-GTP. Dominant-negative Rab11 expressed in the striatum and cortex of normal mice caused neuropathology and motor dysfunction, suggesting that a deficiency in Rab11 activity is pathogenic *in vivo*. Primary cortical neurons from HD140Q/140Q mice were delayed in recycling transferrin receptors back to the plasma membrane. Partial rescue from glutamate induced cell death occurred in HD neurons expressing dominant active Rab11 (22). The polyglutamine expansion in the Htt protein leads to perturbations in many cellular pathways, including the disruption of Rab11-dependent endosomal recycling. Impairment of the small GTPase Rab11 leads to the defective formation of vesicles in HD models and may thus contribute to the early stages of the synaptic dysfunction in this disorder. The expression of mutant htt in the *Drosophila* larval neuromuscular junction decreases the presynaptic vesicle size, reduces quantal amplitudes and evoked synaptic transmission and alters larval crawling behavior. Furthermore, these indicators of early synaptic dysfunction are reversed by the over expression of Rab11 (23). Oxidative stress contributes to neuro-degeneration in HD. Studies in HD transgenic models suggest involvement of mitochondrial dysfunction, which would lead to overproduction of reactive oxygen species (ROS). Impaired mitochondrial complexes occur in late stages of HD but not in presymptomatic or early stage HD patients. Thus, other mechanisms may account for the earliest source of oxidative stress caused by endogenous mutant Htt. Decreased levels of a major intracellular antioxidant glutathione coincide with the accumulation of ROS in primary HD neurons prepared from embryos of HD knock-in mice (HD 140Q/140Q), which have human *huntingtin *exon1 with 140 CAG repeats inserted into the endogenous mouse *huntingtin *gene. Uptake of extracellular cysteine through the glutama-te/cysteine transporter EAAC1 is required for *de novo *synthesis of glutathione in neurons. HD neurons had less numbers of cell surface EAAC1 in comparison to the wild type neurons and were deficient in taking up cysteine. Rab11 mediates constitutive trafficking of EAAC1 from recycling endosomes and this trafficking is defective in the brain of HD 140Q/140Q mice. Dominant-active Rab11 mutant expression in primary HD neurons improved the deficit in cysteine uptake, increased levels of intracellular glutathione, normalized clearance of ROS, and improved neuronal survival and hence support a novel mechanism for oxidative stress in HD. Deregulation of Rab11 function impairs both trafficking of EAAC1 to the cell surface and cysteine uptake, thereby leading to deficient synthesis of glutathione (24). HD is also caused by the increase of a poly glutamine tract in the huntingtin protein (Htt) that mediates the formation of intracellular protein aggregates. Accretion of protein aggregates, in the brains of HD patients and HD transgenic mice, has been coupled to lesions in axo-dendritic and synaptic compartments. Places of synaptogenesis, i.e., dendritic spines are vanished in the closeness of Htt accumulation because of functional defects in Rab11 mediated by local endosomal recycling. In cultured hippocampal neuron cells expressing a mutant Htt fragment, impaired exit from recycling endosomes (RE) and the association of endocytosed protein with intracellular structures containing Htt aggregates was found. Dendrites in hippocampal neurons became dystrophic around enlarged Rab11 positive amphisome-like structures where mutant Htt aggregates. Rab11 over-expression rescues neurodegeneration and significantly extends lifespan in a *Drosophila* model of HD. These findings are consistent with the model that mutant Htt aggregation increases local autophagic activity, thereby sequestering Rab11 and diverting spine-forming cargo from RE into enlarged amphisomes. This mechanism may contribute to the toxicity caused by protein mis-folding found in a number of neurodegenerative diseases (25). This is also caused by a CAG repeat expansion in the huntingtin gene. In the brain of HD patients, a decline in glucose metabolism is observed. Glucose consumption in embryonic primary cortical neurons of wild type (WT) and HD knock-in mice have 140 CAG repeats inserted in the endogenous mouse huntingtin gene; HD (140Q/140Q). Lesser amount of glucose is significantly taken up by primary HD (140Q/140Q) cortical neurons than WT neurons. Glucose uptake in WT neurons is equally altered by expression of permanently inactive and permanently active forms of Rab11 suggesting that normal activity of Rab11 is needed for neuronal uptake of glucose. These results suggest that deficient activity of Rab11 is a novel mechanism for glucose hypometabolism in HD (26). Htt plays an important role in manipulating Rab11 vesicles. Reduction of Htt, kinesin and dynein motors diminished Rab11 vesicle transport indicating a role of these motors and Htt in bidirectional transport of Rab11. Therefore, Htt plays a key role in the movement of Rab11 vesicles within axons and thus, disruption of transport mediated by mutant Htt could contribute to early neuropathology observed in Huntington's diseases (27).

**Type 2 diabetes**

Glucose is uptaken by insulin through translocation of the glucose transporter, GLUT4 to the plasma membrane. In the basal state, GLUT4 interact with cytosolic proteins within various endosomal recycling compartments. Depending on the unambiguous vesicles that GLUT4 travels through, several Rab GTPase family members are vital to this process. In case of type 2 diabetes, insulin resistance disrupts translocation of the intracellular resources of GLUT4 to the plasma membrane, in spite of normal GLUT4 expression. Rab11 is functionally associated with GLUT4 trafficking from storage vesicles to the endocytic recycling pathway (28, 29).

**Cancer**

Studies have indicated that recycling members (Rab11a, Rab11b, Rab25/Rab11c as well as their effectors) exert an important role in cancers of multiple lineages, including breast, colon, lung, ovarian, renal, endometrial, prostate, bladder and carcinoid types (30). It has been shown that Rab25 is involved in cancer progression by cell proliferation and fortification from apoptosis. Its increased expression was seen in ovarian and in breast cancers, where it has been shown to have a key function both in vitro and in vivo cancer progression (31-33). Increased expression was also seen in transitional cell carcinoma of the bladder (Wilms tumour), in prostate cancer (34, 35), in invasive breast tumour cells (33), and in colon cancer cells (36). All of these studies have indicated that it has a pathological role in tumor progression in several epithelial lineages. Investigations have indicated that Rab25 has been implicated in the succession of tumor by regulating the localization of integrin-recycling vesicles to develop tumor invasion. It has also been shown to decrease apoptosis and to increase proliferation as well as aggressiveness of ovarian and breast cancer. Over expression of Rab25 in ovarian cancer cell lines enhances anchorage-independent colony-formation, cell proliferation and cell survival over various stress conditions (37). It has also been associated with the α5β1 integrin enhancing the ability of ovarian cancer cells to invade the extracellular matrix (38). Recently, investigations have shown that Rab25 acts as a tumor suppressor in colon and oesophageal cancers, regulating cell adhesion, polarity and signalling pathways. Further more, research has implicated that an important protein CLIC3 (chloride intracellular channel protein 3) may determine whether or not Rab25 acts as a tumor promoter or suppressor (39-42). It has also been suggested that the expression status of the Rab25 effector RCP could be a determinant of the ability of Rab25 to act as a tumor promoter or suppressor (43). Regulation of cell survival is mediated by anti-apoptotic molecules such as BCL-2, BAX, BAK, and phosphoinositide-3-kinase. Forced expression of Rab25 in ovarian cancer cells reduces the levels of BAX and BAK, and enhances the AKT phosphorylation, which in turn activates the phosphoinositide-3-kinase pathway. Down-regulation of Rab25 reverses apoptotic signalling cascade through BAK and BAX protein signalling as well as AKT activity pathways. The BCL-2 and phosphoinositide-3-kinase pathways are enhanced by Rab25-mediated signaling for cell survival (37, 44-46). In 50% of ovarian cancers, 1q22 amplicon having Rab25 is amplified (32). In addition, chromosome 1q is gained in 50% of breast cancer patients. Rab25 has also been shown to function as a tumor suppressor in hormonally insensitive breast tumors where its expression is lost (47). It may function as a tumor suppressor in intestinal mucosa cells and human colorectal adenocarcinoma where loss of Rab25 leads to increased tumorigenesis through alteration in the regulation of protein trafficking to the cell surface (38). Research has identified that loss of Rab25 expression occurs through a mutation in locus 1q22-23 of human breast cancer tissues. Therefore, Rab25 could be used as a biological marker for breast cancer (48). Another member of Rab11 family, Rab11a, acts as a novel tumor associated c-Fos/AP-1 target, may point to an as yet unrecognized function of Rab11a in the development of skin cancer (49). Rab25 has more frequently been implicated in tumorigenesis than other members of Rab11 family because of the presence of DTAGLE sequence at GTP binding site instead of DTAGQE found in Rab11 as well as in most of the Rab family members. This makes Rab25 constitutively active similar to the Q61L oncogenic mutation in Ras (50).

## Conclusion

Rab11, a master regulator of intracellular membrane trafficking routes, is the most characterized molecule among all the Rab proteins. It has been implicated in the progression of a number of human diseases. It is noteworthy to mention that Rab11 has an extensive protein-protein interactions network and a variety of distinct cargo molecules. But still, several significant questions remain unclear including how Rab11 mediated pathways are regulated; what is the precise molecular mechanism of the upstream activation of Rab11 subfamily members. Indeed, understanding the precise molecular mechanism for Rab11 in membrane recruitment is a key question elucidating its functional role within the cell. A more complete picture of the molecular and cellular events controlled by Rab11 proteins will uncover additional important physiological processes of the Rab11 subfamily members and will prompt further research in molecular mechanisms for many of the diseases in which it has been implicated. Further, understanding how the various interdependent cellular processes occur is now an important goal in the field of membrane trafficking pathways which is yet to be addressed. The members of Rab11 GTPase family are involved in a variety of disease settings including cancer progression. As the actions of mechanisms of these GTPases are identified; the signalling and trafficking pathways in which they operate may present novel targets for therapeutic invention.

## Conflict of interest

The authors declared no conflicts of interest. 
